# Supplementary material for: The Greater Proportion of Born-Light Progeny from Sows Mated in Summer Contributes to Increased Carcass Fatness Observed in Spring
Source: Animals (Basel). 2020 Nov 10;10(11):2080. doi: 10.3390/ani10112080 (PMC7696037; doi:10.3390/ani10112080)
Supplement: Supplementary file 1 [file animals-10-02080-s001.pdf]

Article

The Greater Proportion of Born-Light Progeny from Sows Mated in Summer Contributes to Increased Carcass Fatness Observed in Spring

Fan Liu [1,*], Erin M. Ford [1], Rebecca S. Morrison [1], Chris J. Brewster [1], David J. Henman [1], Robert J. Smits [1,†], Weicheng Zhao [2], Jeremy J. Cottrell [2], Brian J. Leury [2], Frank R. Dunshea [2,3] and Alan W. Bell [4]

[1] Rivalea Australia Pty Ltd., Corowa, NSW 2646, Australia; fliu@rivalea.com.au (F.L.); eford@rivalea.com.au (E.M.F.); rmorrison@rivalea.com.au (R.S.M.), cbrewster@rivalea.com.au (C.J.B.); dhenman@rivalea.com.au (D.J.H.); rsmits@rivalea.com.au or rob.smits@australianpork.com.au (R.J.S.)

[2] Faculty of Veterinary and Agricultural Sciences, University of Melbourne, Parkville 3010, Australia; weichengz@student.unimelb.edu.au (W.Z.); jcottrell@unimelb.edu.au (J.J.C.), brianjl@unimelb.edu.au (B.J.L.); fdunshea@unimelb.edu.au (F.R.D.)

[3] Faculty of Biological Sciences, The University of Leeds, Leeds LS2 9JT, UK

[4] Department of Animal Science, Cornell University, Ithaca, NY 14853, USA; alanwilliambell@gmail.com

* Correspondence: fliu@rivalea.com.au; Tel.: +61-3-6033-8265

† Current address: Australian Pork Limited, Barton, ACT 2600, Australia

Received: 29 October 2020; Accepted: 07 November 2020; Published: 10 November 2020

Table S1. Composition of experimental diets

Nutrients	Gestation diet	Weaner diet	Grower diet	Finisher diet
Digestible energy, MJ/kg	13.1	14.6	13.9	13.5
Crude protein, %	13.0	20.7	17.2	14.7
available Lysine, %	0.52	1.31	0.97	0.81
Calcium, %	1.00	1.38	0.99	0.98
available phosphorus, %	0.44	1.1	0.44	0.40

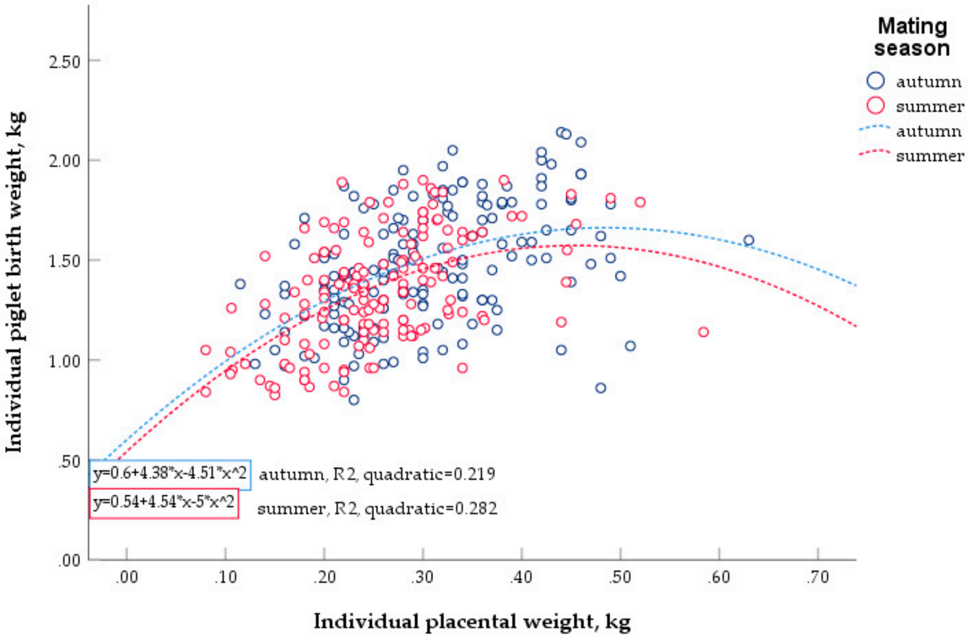

Figure S1. Relationship between placental weight and birth weight.
